# Evaluation of sensitivity and specificity of Kato-Katz and circulating cathodic antigen in terms of *Schistosoma japonicum* using latent class analysis

**DOI:** 10.1038/s41598-024-57863-9

**Published:** 2024-04-08

**Authors:** Mugyeom Moon, Hannah W. Wu, Mario Jiz, Stephanie Maldonado, Jonathan D. Kurtis, Jennifer F. Friedman, Blanca Jarilla, Sangshin Park

**Affiliations:** 1 Food and Agriculture Organization of the United Nations, Regional Office for Asia and Pacific, Bangkok, Thailand; 2 The Warren Alpert Medical School of Brown University, Providence, RI USA; 3 Center for International Health Research, Rhode Island Hospital, The Warren Alpert Medical School of Brown University, Providence, RI USA; 4 Department of Pediatrics, Rhode Island Hospital, The Warren Alpert Medical School of Brown University, Providence, RI USA; 5 Department of Health, Research Institute for Tropical Medicine, Manila, Philippines; 6 Department of Pathology and Laboratory Medicine, The Warren Alpert Medical School of Brown University, Providence, RI USA; 7 Graduate School of Urban Public Health, University of Seoul, Seoul, Republic of Korea

**Keywords:** Parasitology, Medical research

## Abstract

*Schistosoma japonicum* is endemic in the Philippines. The Kato-Katz (KK) method was used to diagnose *S. japonicum*. This is impractical, particularly when the sample size is limited. Knowledge on point-of-care circulating cathodic antigen (CCA) test performance for *S. japonicum* is limited. Determining the sensitivity and specificity of new diagnostics is difficult when the gold standard test is less effective or absent. Latent class analysis (LCA) can address some limitations. A total of 484 children and 572 adults from the Philippines were screened for *S. japonicum*. We performed Bayesian LCA to estimate the infection prevalence, sensitivity and specificity of each test by stratifying them into two age groups. Observed prevalence assessed by KK was 50.2% and 31.8%, and by CCA was 89.9% and 66.8%, respectively. Using Bayesian LCA, among children, the sensitivity and specificity of CCA were 94.8% (88.7–99.4) and 21.5% (10.5–36.1) while those of KK were 66.0% (54.2–83.3) and 78.1% (61.1–91.3). Among adults, the sensitivity and specificity of CCA were 86.4% (76.6–96.9) and 62.8% (49.1–81.1) while those of KK were 43.6% (35.1–53.9) and 85.5% (75.8–94.6). Overall, CCA was more sensitive than KK, regardless of the age group at diagnosis, as KK was more specific. KK and CCA have different diagnostic performance, which should inform their use in the planning and implementation of *S. japonicum* control programs.

## Introduction

Schistosomiasis is a neglected tropical disease that causes significant morbidity including anemia, undernutrition, and neurocognitive delays among children^1^, lower work capacity among adults^2^ and end-organ damage in a subset of chronically infected individuals. It is estimated that approximately 240 million people worldwide are at risk and approximately 200,000 people die annually from schistosomiasis. Six species of *Schistosoma* have been distributed across Africa, the Middle East, Asia, and South America^3^. *Schistosoma japonicum* is endemic in China, Indonesia, and the Philippines^4^ with the highest proportion in the Philippines, where the prevalence of *S. japonicum* varies by region with approximately 2.5 million people at risk across the country^5^.

Currently, there is no gold standard test for the diagnosis of intestinal schistosomiasis. Kato-Katz (KK) and point-of-care circulating cathodic antigen (CCA) are used with stool and urine samples. KK has been widely used as a standard test and is recommended by the World Health Organization^6,7^, however, KK has poor sensitivity in low-level infection settings^8^, and multiple repeat assessments on multiple collected stool samples are needed to maximize sensitivity. This makes it incredibly cumbersome to use in low- and middle-income countries. Given the difficulty of this diagnostic approach and the fact that a single dose of praziquantel is highly effective, globally, mass drug administration (MDA) is employed in most settings, whereby individuals are offered treatment without assessing the presence of infection.

The CCA, a point-of-care urine test, has been shown to be more sensitive than KK in the context of *S. mansoni*^9–18^. Little is known about the performance of CCA in *S. japonicum* in the absence of gold standard*.* In addition to increased sensitivity, ease of use presents another advantage as individuals can be diagnosed quickly in endemic villages during MDA programs to identify those in need of treatment. Some serological test methods such as enzyme-linked immunosorbent assay (ELISA), indirect hemagglutination assay (IHA), and polymerase chain reaction (PCR) show higher sensitivity^19^. However, when dealing with large size population especially in the field settings, if more sensitive and easy-to-use tests were available, it would be easier to estimate community prevalence. The WHO recommends that community prevalence estimates should inform the frequency of treatment for MDA programs because a commonly reported reason for refusal of MDA is the lack of knowledge of infection status^20,21^.

As in the case of schistosomiasis^9–11^, the absence of a gold standard makes it difficult to assess new diagnostics because each applied test produces a certain number of “incorrect” diagnoses. Latent class analysis (LCA) is a useful method for identifying latent disease cases in the absence of a gold standard test^22^. The LCA has been used to assess the performance of CCA and KK in determining the prevalence of *S. mansoni*^9–18^*,* however, no studies have attempted to assess the performance of CCA and KK using LCA in *S. japonicum.* This study employed LCA to determine the sensitivity and specificity of KK and point-of-care CCA methods for the diagnosis of *S. japonicum*, which will contribute to the planning, implementing, and evaluating schistosomiasis control programs.

## Methods

### Ethical statement

This study was approved by the institutional review boards of Rhode Island Hospital (Providence, RI, USA) and the Research Institute of Tropical Medicine, Manilla, The Philippines. All participants over the age of 18 provided an informed written consent. Consent was obtained in private spaces in the native language Waray. All consent forms were translated into Waray and back translated for accuracy review. For non-literate subjects, a witnessed verbal consent process was used. For children aged eight or above, both their assent and parental written informed consent were obtained while for children under the age of eight, written informed consent from legally authorized representatives/guardians was obtained. This study followed the Ethical Principles for Medical Research Involving Human Subjects. All procedures were performed in accordance with relevant guidelines.

### Study population

The study was carried out in an *S. japonicum* endemic rice-farming village (Barangy Macanip) in the Municipality of Jaro in Leyte, Philippines. *S. japonicum* is endemic in Leyte due to rice farming and contact with several streams and rivers for activities of daily living being the primary source of exposure. The MDA is delivered at the community level annually to individuals over four years of age.

**Figure 1:**
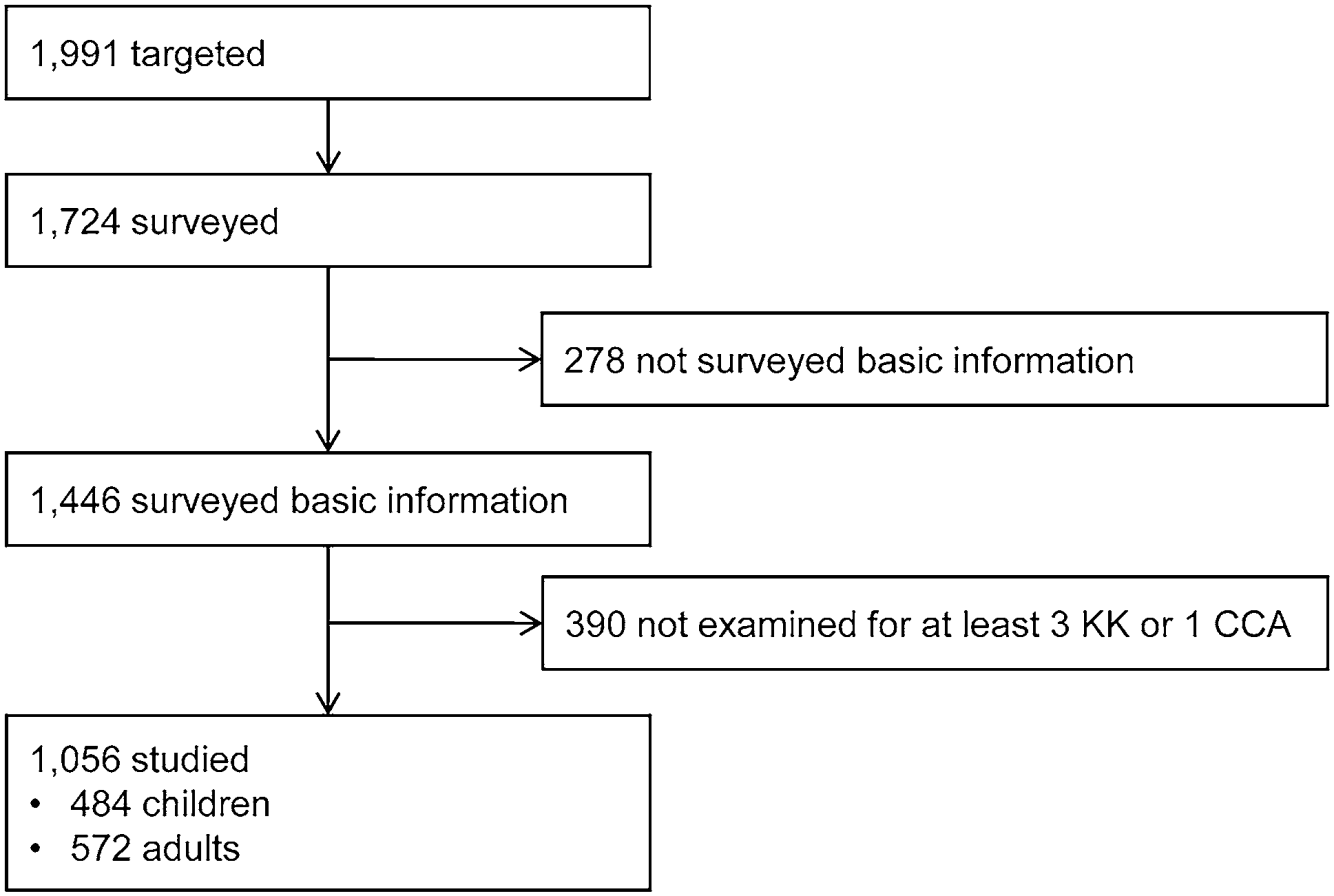
Flow chart of study participants.

The village population was 1991 and consisted of 12 sitios (geographic zones) and 451 households. The age range of the population was from newborns to 90 years with an average age of 27 years. Mapping and census were conducted in February 2016. The stool survey and CCA screening were performed from April 2016 to February 2017 after informed consent was obtained. A total of 1056 participants (484 children and 572 adults) were included in this study (Fig. 1). The age range of the children was from newborns to 19 years old, including 267 children under 7 years old, whereas that of adults was ranged from 20 to 87 years.

### Sampling procedure and laboratory analysis

For all the participants included in this study, schistosomiasis infection was assessed by Kato-Katz (KK) thick smears technique. Briefly, each fecal sample was pressed through a sieve, and the amount of 50 mg sieved stool measured by a standard template was transferred to a microscope slide where a piece of cellophane soaked in glycerine was pressed on the sample. Two slides were made from each of the three stool samples collected on separate days. For each of the stool specimens, the average number of eggs per gram of the duplicate test was determined, and the overall mean eggs per gram was derived by averaging the parasite burden of the 3 individual specimens. Slides were read by two trained medical technologists who have performed thousands of KK tests, with initial teaching and review of slides by a more experienced technologist. Each slide was read by one technologist; however, two different technologists could have read three different stools per participant. For quality control, an independent expert microscopist will randomly examine 10% of all slides to assess the quality of field diagnosis.

In addition, we performed a point-of-care CCA test (POC-CCA assay, Rapid Medical Diagnostics Pretoria, South Africa, sourced from Maternova) on a single urine sample within one week of the KK assessment. The test kits were kept dry within the temperature of 4–28 °C. The urine samples were not stored, and the test was performed at the time of sample collection. The urine samples were collected during separate field trips to collect stool samples, but all samples were collected within a week of the final stool collection. A participant was diagnosed as CCA positive when the control band turned pink, and a band was present in the test area of CCA. Trace results were regarded as positive according to the test kit manual from the manufacturer, meaning that all trace results were recorded as positive. Therefore, all test results were recorded as either positive or negative.

All the participants aged four and over with schistosomiasis as determined by KK results were treated with a single 40 mg/kg dose of Praziquantel. At the time of the study, praziquantel was not approved by the Philippines Department of Health for children under the age of four. The CCA was performed before the treatment. Treatment with a single dose of albendazole was also provided to all participants over the age of two with Soil-transmitted helminths (STH) identified by KK as per the Philippines Department of Health Guidelines at the time.

### Statistical analysis

We performed Bayesian LCA to estimate the infection prevalence, sensitivity, and specificity of each test using OpenBUGS version 3.2.3 rev 1012 software (Members of OpenBUGS Project Management Group) through the R2OpenBugs package^23^ in R version 4.0.3 software^24^. We stratified the analysis into two age groups, children and adults (ages 18 and over) (Supplementary Methods).

*1. Model description for Bayesian LCA:* As the results of the KK and CCA tests reflect a common biological phenomenon, we considered that the two test results could be conditionally dependent^25^. As proposed by Dendukuri and Joseph^26^, we fitted the models while adjusting for conditional dependence between the results of the KK and CCA tests. Depending on the status of the disease, the probabilities observing combinations of test results can be specified with the prevalence (prev_*i*_) of *i*th subpopulation, sensitivity (Se) and specificity (Sp) of the tests, and the covariance in sensitivity (covSe) and specificity (covSp) between two tests. The probabilities (P_*i*_) of observing each test combination in each subpopulation would become:

$$\begin{aligned} {\text{P}}_{i} \left( { +_{{{\text{KK}}}} , \, +_{{{\text{CCA}}}} } \right) \, & = {\text{ prev}}_{i} \times \, \left( {{\text{Se}}_{{{\text{KK}}}} \times {\text{ Se}}_{{{\text{CCA}}}} + {\text{ covSe}}} \right) \, + \, \left( {{1 }{-}{\text{ prev}}_{i} } \right) \, \times \, \left[ {\left( {{1 }{-}{\text{ Sp}}_{{{\text{KK}}}} } \right) \, \times \, \left( {{1 }{-}{\text{ Sp}}_{{{\text{CCA}}}} } \right) \, + {\text{ covSp}}} \right], \\ {\text{P}}_{i} \left( { +_{{{\text{KK}}}} , \, {-}_{{{\text{CCA}}}} } \right) \, & = {\text{ prev}}_{i} \times \, \left[ {{\text{Se}}_{{{\text{KK}}}} \times \, \left( {{1 }{-}{\text{ Se}}_{{{\text{CCA}}}} } \right) \, {-}{\text{ covSe}}} \right] \, + \, \left( {{1 }{-}{\text{ prev}}_{i} } \right) \, \times \, \left[ {\left( {{1 }{-}{\text{ Sp}}_{{{\text{KK}}}} } \right) \, \times {\text{ Sp}}_{{{\text{CCA}}}} {-}{\text{ covSp}}} \right], \\ {\text{P}}_{i} \left( {{-}_{{{\text{KK}}}} , \, +_{{{\text{CCA}}}} } \right) \, & = {\text{ prev}}_{i} \times \, \left[ {\left( {{1 }{-}{\text{ Se}}_{{{\text{KK}}}} } \right) \, \times {\text{ Se}}_{{{\text{CCA}}}} {-}{\text{ covSe}}} \right] \, + \, \left( {{1 }{-}{\text{ prev}}_{i} } \right) \, \times \, \left[ {{\text{Sp}}_{{{\text{KK}}}} \times \, \left( {{1 }{-}{\text{ Sp}}_{{{\text{CCA}}}} } \right) \, {-}{\text{ covSp}}} \right], \\ {\text{P}}_{i} \left( {{-}_{{{\text{KK}}}} , \, {-}_{{{\text{CCA}}}} } \right) \, & = {\text{ prev}}_{i} \times \, \left[ {\left( {{1 }{-}{\text{ Se}}_{{{\text{KK}}}} } \right) \, \times \, \left( {{1 }{-}{\text{ Se}}_{{{\text{CCA}}}} } \right) \, + {\text{ covSe}}} \right] \, + \, \left( {{1 }{-}{\text{ prev}}_{i} } \right) \, \times \, \left( {{\text{Sp}}_{{{\text{KK}}}} \times {\text{ Sp}}_{{{\text{CCA}}}} + {\text{ covSp}}} \right). \\ \end{aligned}$$

We limited the covariances as proposed by Dendukuri and Joseph^24^:

$$\begin{gathered} \left( {{\text{Se}}_{{{\text{KK}}}} - { 1}} \right) \, \times \, \left( {{1 }{-}{\text{ Se}}_{{{\text{CCA}}}} } \right) \, \le {\text{ covSe }} \le {\text{ min }}\left( {{\text{Se}}_{{{\text{KK}}}} ,{\text{ Se}}_{{{\text{CCA}}}} } \right) \, - {\text{ Se}}_{{{\text{KK}}}} \times {\text{ Se}}_{{{\text{CCA}}}} , \hfill \\ \left( {{\text{Sp}}_{{{\text{KK}}}} - { 1}} \right) \, \times \, \left( {{1 }{-}{\text{ Sp}}_{{{\text{CCA}}}} } \right) \, \le {\text{ covSp }} \le {\text{ min }}\left( {{\text{Sp}}_{{{\text{KK}}}} ,{\text{ Sp}}_{{{\text{CCA}}}} } \right) \, - {\text{ Sp}}_{{{\text{KK}}}} \times {\text{ Sp}}_{{{\text{CCA}}}} . \hfill \\ \end{gathered}$$

We then linked the likelihood functions to the combinations of the observed test results using a multinomial distribution. The probabilities (P_*i*_) were computed separately for children and adults using the same equations.

*2. Estimation of sensitivity and specificity of each test and infection prevalence:* As there was insufficient previous diagnostic research comparing KK and CCA for *S. japonicum*, we implemented a weakly informative distribution, beta (1, 1), as a prior distribution for the infection prevalence of *S. japonicum*^27^*.* Beta (3.05, 1.15) and beta (21.2, 2.06) were adopted as prior distributions of the sensitivity and specificity of the KK technique, respectively, and beta (3.05, 1.15) and beta (5.38, 1.49) were selected as prior distribution of the sensitivity and specificity of the CCA test as suggested by Clements^28^. The model was run with three Markov Chain Monte Carlo (MCMC) chains separately for children and adults. Each parallel chain was run for 12,000 iterations, including an initial burn-in period of 2000 iterations, with a thin of 25^23^. Corresponding to the assumption of prior distributions, initial numeric values of parameters: prev_*i*_, Se_KK_, Sp_KK_, Se_CCA_, and Sp_CCA_ for Bayesian LCA were randomly drawn. We examined the convergence of the model using trace plots^29^, potential scale reduction factor^30^, CODA package^31^ for Gelman-Rubin diagnostic statistics, and shrink factors. Mean and 95% highest posterior density regions of these five parameters were reported for the joint posterior distribution^27^. The estimated infection prevalence within the subpopulation was used to compute the overall infection prevalence according to the number of participants in the test area.

*3. Estimation of test prevalence, positive predictive value (PPV), and negative predictive value (NPV):* Distribution of test prevalence by the KK and CCA tests was obtained separately for children and adults from 30,000 times of binomial trials (an equal number of iterations in the joint posterior distribution) with the total number of participants (children or adults) and overall observed prevalence. The PPV and NPV in children and adults for the KK and CCA tests were computed using the estimated overall infection prevalence, sensitivity, and specificity of each test applying the following equations: To get the distribution of PPV and NPV, in the same way, we conducted 30,000 times of binomial trials. We reported the mean and 95% of percentiles of test prevalence, PPV, and NPV.

$${PPV}_{m,n}= \frac{{prev}_{m,n}\times {Sensitivity}_{m,n}}{{prev}_{m,n}\times {Sensitivity}_{m,n}+{(1-prev}_{m,n})\times {(1-Specificity}_{m,n})}$$

$${NPV}_{m,n}= \frac{(1-{prev}_{m,n})\times {Specificity}_{m,n}}{{prev}_{m,n}\times (1-{Sensitivity}_{m,n})+{(1-prev}_{m,n})\times {Specificity}_{m,n}}$$

(prev denotes overall infection prevalence, m and n denote each test and age group, respectively.)

*4. Comparison of prevalence, sensitivity, specificity, PPV and NPV:* The difference between infection prevalence and test prevalence was computed for comparison. The values of overall infection prevalence in the posterior distribution were subtracted by the values of the test prevalence distribution obtained from 30,000 times of binomial trials. Values of the KK test prevalence distribution were subtracted by the values of the CCA test prevalence distribution to obtain the difference between the two test prevalences. Likewise, the sensitivity and specificity of each test were compared by subtracting each value from its posterior distribution. The PPV and NPV were compared by subtracting each value obtained from the binomial distributions. A comparison of the studied parameters in children and adults was conducted, in the same manner, by subtracting the relevant values.

### Ethics approval and consent to participate

This study was approved by the institutional review boards of Rhode Island Hospital (Providence, RI, USA) and the Research Institute of Tropical Medicine, Manilla, The Philippines. All participants over the age of 18 provided an informed written consent. Consent was obtained in private spaces in the native language Waray. All consent forms were translated into Waray and back translated for accuracy review. For non-literate subjects, a witnessed verbal consent process was used. For children aged eight or above, both their assent and parental written informed consent were obtained while for children under the age of eight, written informed consent from legally authorized representatives/guardians was obtained. This study followed the Ethical Principles for Medical Research Involving Human Subjects. All procedures were performed in accordance with relevant guidelines.

### Consent for publication

During the baseline investigation, all participants were informed before the surveys or interviews that the data collected from them would be used for research and publication purposes. They were assured of the confidentiality of their data and were given the right to withdraw their participation at any time.

## Results

### Descriptive results

The prevalence examined by CCA was higher than that examined by KK, regardless of the age group (Table 1). The prevalence of *S. japonicum* by KK technique in children (50.2%) was higher than that in adults (31.8%). The mean infection intensity of all participants was 24.7 and 9.9 eggs per gram (EPG) in children and adults, respectively. Likewise, the prevalence of *S. japonicum* by CCA in children (89.9%) was higher than that in adults (66.8%). The CCA test was not able to detect 5.2% of children and 8.0% of adults who had positive KK test results, whereas KK did not identify as positive 44.8% of children and 43.0% of adults whose results by CCA were positive.

**Table 1:** Descriptive statistics of study population (n = 1056).

Variables	Children				Adults			
Number of study participants	484				572			
Age range (mean)	0–19 years old (7.7 years old)				20–87 years old (48.1 years old)			
Sex: female, %	47.1				53.0			
*Schistosoma japonicum*								
KK								
Observed prevalence, %	50.2				31.8			
*S. japonicum*, epg	24.7 ± 65.8				9.9 ± 29.5			
CCA								
Observed prevalence, %	89.9				66.8			

			KK				KK	
			Positive	Negative			Positive	Negative
Two test results, n(%)	CCA	Positive	218 (45.0%)	217 (44.8%)	CCA	Positive	136 (23.8%)	246 (43.0%)
		Negative	25 (5.2%)	24 (5.0%)		Negative	46 (8.0%)	144 (25.2%)

### Result of Bayesian LCA

**Figure 2:**
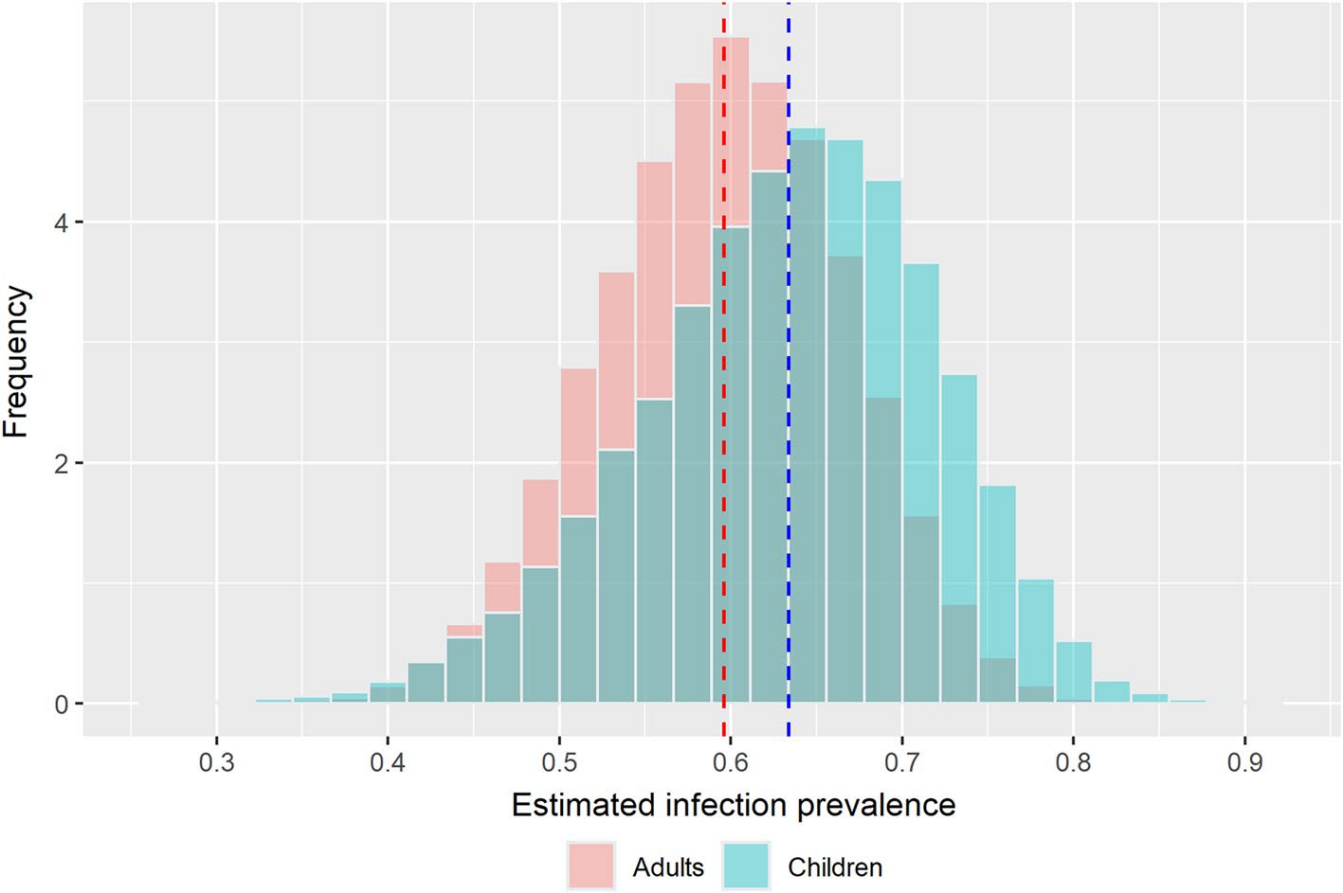
Weighted overall infection prevalence.

The convergence of the Bayesian LCA with three chains was assured from the potential scale reduction factor, trace plots, and Gelman-Rubin statistics. The potential scale reduction factors were less than 1.1 and the shrink factors headed to 1.0 and recorded as less than 1.01. The effective sample sizes for all the estimated parameters ranged from 5000 to 30,000. Overall infection prevalence weighted by the number of participants within each sitio was 63.4% and 59.6% in children and adults, respectively (Fig. 2 and Table 2). The discrepancy between the two estimated means of overall prevalence in both age groups was less than 4% but the 95% highest posterior density region in children was slightly wider than in adults.

**Table 2:** Estimated infection prevalence, sensitivity, and specificity (mean and 95% Bayesian credible interval) from Bayesian LCA and relevant parameters estimated from posterior distributions of Bayesian LCA.

	Children (%)	Adults (%)	Children–adults*
Infection prevalence	63.4 (44.9 to 78.2)	59.6 (45.3 to 72.9)	3.8 (− 18.6 to 24.7)
Sensitivity			
KK	66.0 (54.2 to 83.3)	43.6 (35.1 to 53.9)	22.4 (6.6 to 41.4)
CCA	94.8 (88.7 to 99.4)	86.4 (76.6 to 96.9)	8.4 (− 3.4 to 19.6)
KK-CCA	− 28.8 (− 41.3 to − 11.4)	− 42.7 (− 55.7 to − 30.2)	
Specificity			
KK	78.1 (61.1 to 91.3)	85.5 (75.8 to 94.6)	− 7.3 (− 26.7 to 9.2)
CCA	21.5 (10.5 to 36.1)	62.8 (49.1 to 81.1)	− 41.3 (− 62.7 to − 21.4)
KK-CCA	56.6 (36.3 to 75.4)	22.7 (1.0 to 39.4)	
Test prevalence			
KK	50.2 (45.7 to 54.8)	31.8 (28.0 to 35.7)	18.4 (12.5 to 24.3)
CCA	89.9 (87.2 to 92.6)	66.8 (62.9 to 70.6)	23.1 (18.4 to 27.8)
Comparison of prevalence estimates			
Infection prevalence—KK test prevalence	13.1 (− 5.7 to 28.6)	27.8 (13.1 to 41.6)	
Infection prevalence—CCA test prevalence	− 26.5 (− 45.0 to − 11.4)	− 7.2 (− 22.1 to 6.6)	
KK test prevalence—CCA test prevalence	− 39.7 (− 44.8 to − 34.3)	− 35.0 (− 40.4 to − 29.5)	
PPV			
KK	83.9 (80.6 to 87.2)	81.6 (78.3 to 84.6)	2.3 (− 2.3 to 6.9)
CCA	67.6 (63.4 to 71.7)	77.4 (74.0 to 80.8)	− 9.7 (− 15.2 to − 4.4)
KK-CCA	16.3 (11.0 to 21.5)	4.2 (− 0.3 to 8.9)	
NPV			
KK	57.1 (52.7 to 61.4)	50.7 (46.7 to 54.9)	6.3 (0.2 to 12.4)
CCA	70.5 (66.3 to 74.6)	75.8 (72.2 to 79.2)	− 5.3 (− 10.7 to 0.1)
KK-CCA	− 13.4 (− 19.4 to − 7.4)	− 25.1 (− 30.4 to − 19.6)	

*This indicates the difference of estimates between children and adult group.

**Figure 3:**
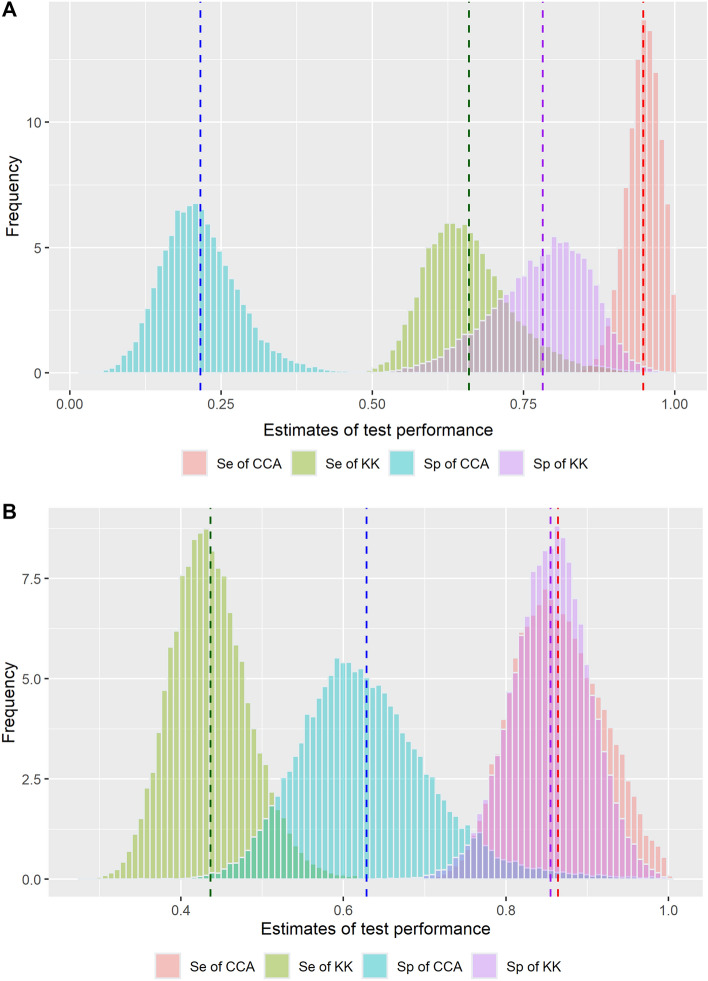
Posterior distribution of sensitivity and specificity with regards to KK method and a point-of-care CCA test [(**A**) children, (**B**) adults].

Sensitivity estimates by CCA were higher than those by KK regardless of the age group (Fig. 3A,B; Table 2). Moreover, sensitivity estimates of each test (KK and CCA) were higher in children (66.0%, 94.8%) than in adults (43.6%, 86.4%). The difference in sensitivity estimates between the two age groups in each test was higher for KK than for CCA.

With respect to specificity, estimates by KK were higher than those by CCA, regardless of age group (Fig. 3 and Table 2). The adult age group showed higher specificity than the group of children, regardless of test methods; specificity: 85.5% (KK) and 62.8% (CCA) in the adult group, 78.1% (KK) and 21.5% (CCA) in children group, respectively. With respect to specificity estimates differences between age groups, the difference by CCA (62.8% vs. 21.5% among adults vs. children) was higher than that by KK (85.5% vs. 78.1% among adults vs. children).

### Comparison of infection prevalence and test prevalence

**Figure 4:**
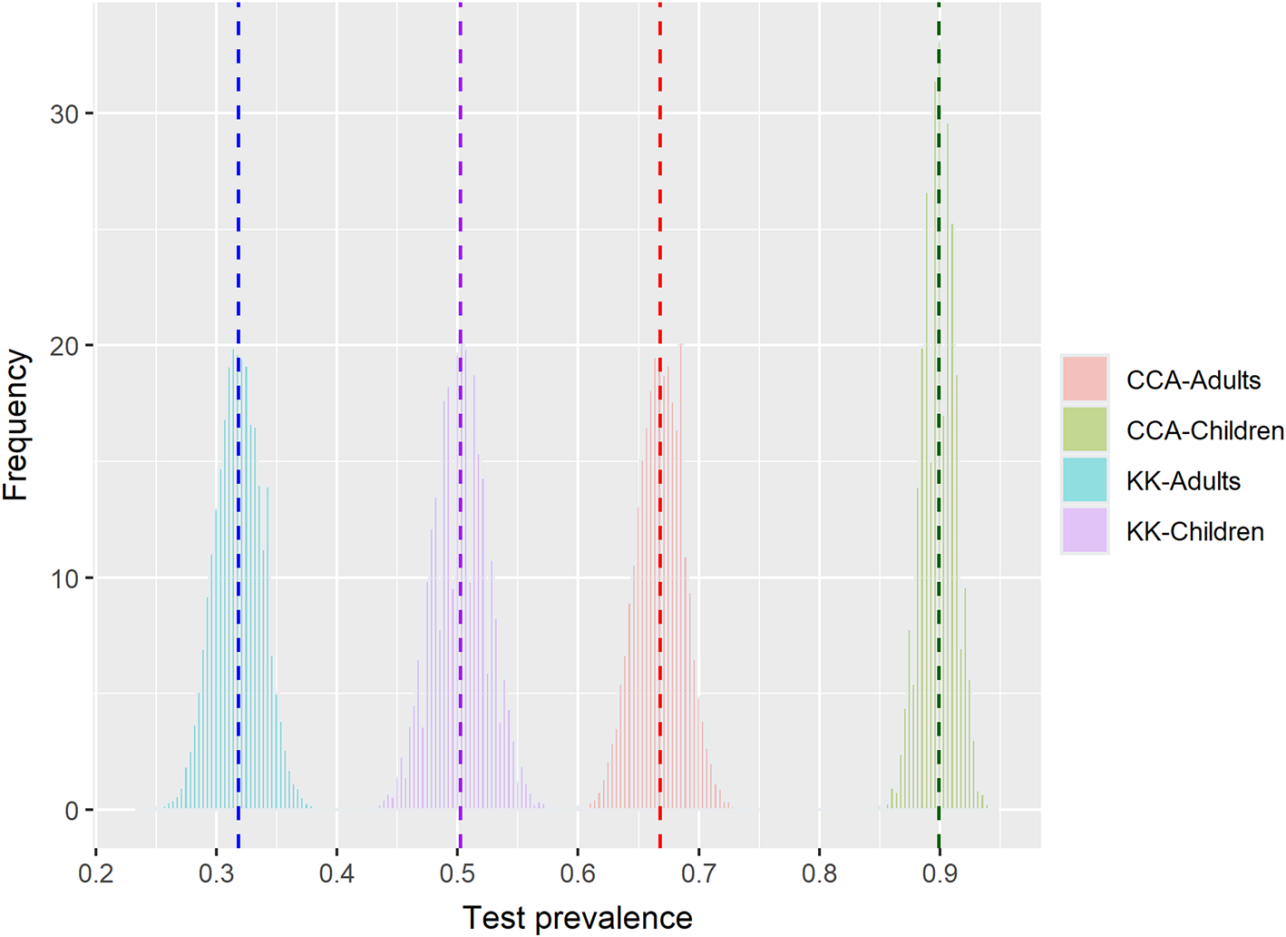
Distribution of test prevalence by diagnostic test method and by age group.

Test prevalence is the expected prevalence from binomial trials rather than the observed prevalence. Test prevalence estimates differed according to the test methods and age groups (Fig. 4 and Table 2). KK underestimated the prevalence of infection, whereas the CCA overestimated it. To elaborate, KK test prevalence (children: 50.2% and adults: 31.8%) was lower than infection prevalence, whereas the CCA test prevalence (children: 89.9% and adults: 66.8%) was higher than both estimated infection prevalence and KK test prevalence.

The two test prevalences and estimated infection prevalence were compared. The differences were small when comparing infection prevalence with the KK test prevalence among children and when comparing infection prevalence with the CCA test prevalence among adults (Table 2). As KK and CCA under- and over-estimated the infection prevalence respectively, the difference between the two test prevalences was higher than any difference in the prevalence between infection and each individual test prevalence.

### Results of test PPV and NPV

**Figure 5:**
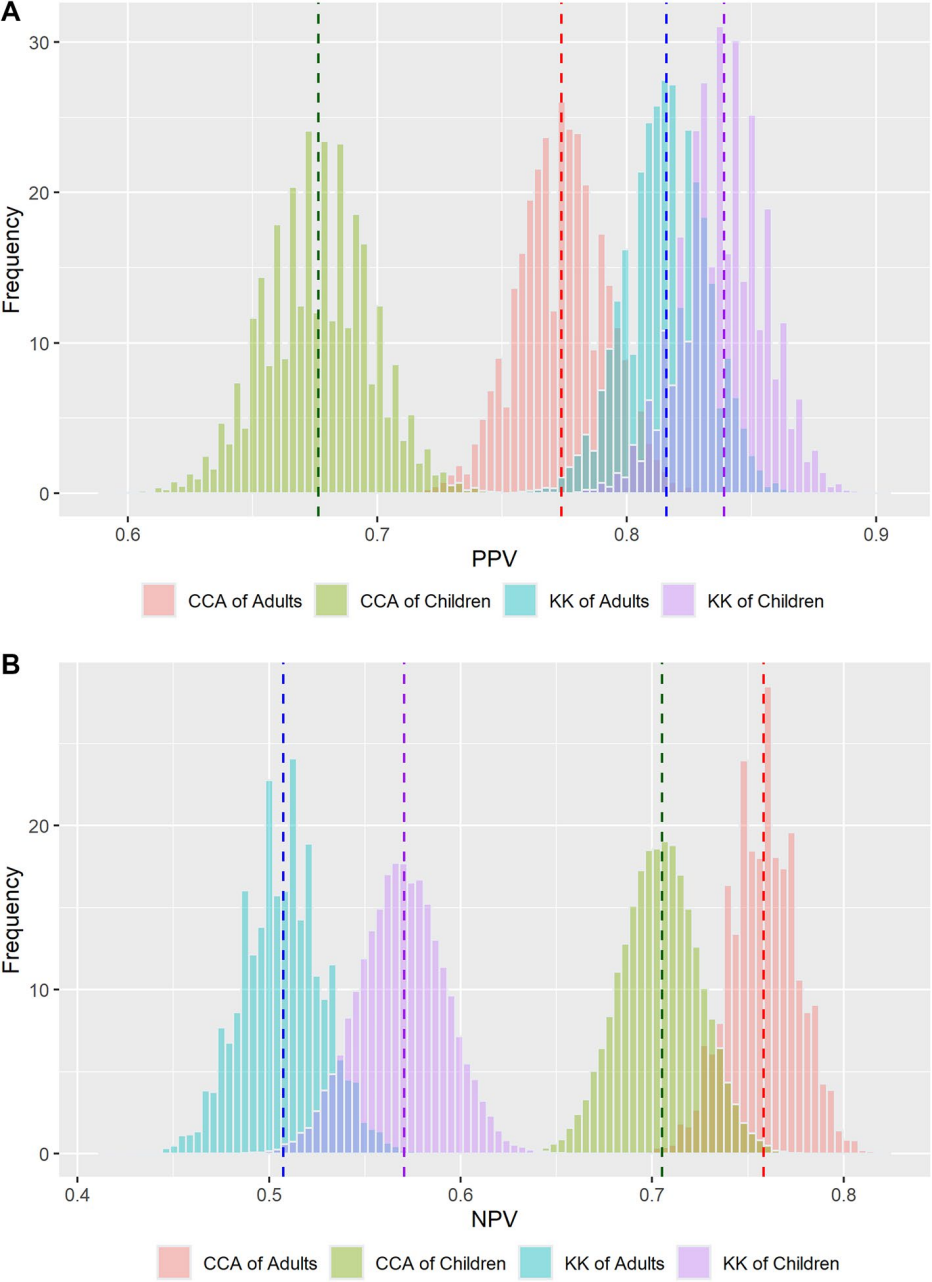
Distribution of (**A**) PPV and (**B**) NPV by diagnostic test method and by age group.

The PPV and NPV values obtained using the same test method were similar across age groups (Fig. 5A,B; Table 2). The PPV mean values from KK (children: 83.9% and adults: 81.6%) were higher than those from CCA (children: 67.6% and adults: 77.4%). On the other hand, NPV mean values from CCA (children: 70.5% and adults: 75.8%) were higher than those from KK (children: 57.1% and adults: 50.7%).

## Discussion

The principal findings of this study include the estimation of infection prevalence of *S. japonicum* in Leyte, the Philippines, and estimations of sensitivity and specificity of the KK technique and CCA test by integrating the disagreement between the two test methods’ results within a framework of LCA^32^. To the best of our knowledge, no previous study has carefully evaluated CCA’s performance in the context of *S. japonicum*, particularly with more sophisticated statistical approaches. It is critical to be able to diagnose individuals accurately and to estimate community-level prevalence in order to guide the frequency of MDA treatment as outlined by the WHO. In addition, identifying point-of-care tests that can be easily deployed in field settings is of great public health importance, because many individuals in endemic areas refuse MDA treatment without assessment of the presence of infection^20,21^. The KK method requires the expertise of a medical technologist, substantial amount of time, and multiple stools collected on different days to maximize sensitivity^33^. Concerning *S. japonicum*, despite the fact of low sensitivity, especially in low-intensity endemic areas, KK is still used as the only gold standard test, whereas in *S. mansoni* transmission areas, the CCA test can also be used for people living the endemic fields^1^. Therefore, we employed LCA to evaluate the test performances of KK and CCA for the diagnosis of *S. japonicum* of study participants from the villages in the Philippines^34^.

A series of studies have evaluated the test performances of CCA and KK as well as the infection prevalence in *S. mansoni* using LCA^10,28^, despite a lack of previous research on *S. japonicum*. Some LCA models incorporated test results not only from KK and CCA but also from other methods such as ELISA^11,12^, polymerase chain reaction (PCR)^12–15^, circulating anodic antigen (CAA)^9,16^, DNA TaqMan^17^, and loop-mediated isothermal amplification (LAMP)^12^ together.

This study employed the LCA model proposed by Dendukuri and Joseph^26^. A more robust LCA model was proposed by Clement^28^ which was derived to consider trace results as positive and negative, separately. Clement’s study^28^ presented higher sensitivity and lower specificity when all trace results were considered positive than when all trace results were considered negative. As the current study considered all trace results as positive, we used the LCA model proposed by Dendukuri and Joseph^26^.

This study stratified the analyses by age group to understand how test performance varied according to the participant age, as demonstrated in another study^35^. Thus, infection prevalence was estimated separately for children and adults, and both prevalences were consistent. In particular, the prevalence of infection among children under the age of 7 years old in the Philippines was 50.7%, which is important as this age group has not been previously assessed for prevalence to our knowledge in the Philippines.

The overall estimated sensitivity of CCA was higher than KK, reflecting the proportion of discordant pairs with more than 43% CCA positive and KK negative and less than 10% CCA negative and KK positive across all ages. This has been demonstrated in other studies that simply compared CCA and KK^36^ and applied LCA^9–18^. This was also true in our study, which collected three stool samples for KK rather than two, as is often assessed. The higher difference in sensitivity between the two test methods in the adults compared to children arose from the lower percentage of positive concordant pairs in the adult group (23.8%) vs. the child group (45.0%). Moreover, the high CCA test sensitivity compared to KK in both age groups implies that CCA is more likely to accurately identify individuals with *S. japonicum* by finding a greater number of true positives regardless of participants’ age even when three KK were evaluated. Given that there are few side effects of treatment with great benefits, enhanced sensitivity is the ideal approach in most settings; however, its widespread use is limited by cost. The estimated sensitivity of CCA from both age groups on *S. japonicum* was higher^10,13, 15, 28^ or lower^11,14, 17^ than that from previous studies on *S. mansoni*. Although the range of sensitivity of serological tests varied depending on the serological techniques and antigens, some serological tests such as ELISA, western blot (WB), electroimmunotransfer blot (EITB), IHA, and immunoblot (IB) against certain antigens showed higher and constant sensitivity^37^ compared to the CCA sensitivity estimated in this study.

The estimated specificity by KK was higher than that estimated by CCA, regardless of the age group, indicating that KK would find true negatives for *S. japonicum* with high credibility. This is likely due to the appearance characteristic of Schistosoma eggs by microscopy. Likewise, KK showed a higher specificity than CCA in other LCA studies^10–12, 14–18, 28^. Moreover, recent studies have raised concerns that CCA may have low specificity. In particular, CCA positive results were demonstrated in a no to low endemicity area of Brazil with positive CCA tests noted among individuals with negative KK and Helmintex tests^38^. Similar studies conducted among individuals living in non-exposed European countries have raised similar concerns regarding false-positive test results by CCA, including the relationship between urine acidity and test positivity^35^.

In particular, among children, the CCA test demonstrated higher sensitivity and lower specificity than KK. This aligns with other studies showing that CCA has a higher sensitivity than KK^9–11, 13–18^. In addition, the aforementioned study in the Netherlands^35^ demonstrated a particularly low specificity among young children. The reasons for the difference in children may be due to low urine pH, and differences in specific gravity (concentration) compared to adults, hematuria, and urine leukocytes, as demonstrated in some studies^39–41^.

The PPV of KK was higher than that of CCA, regardless of the age group. This could be because of the low specificity of CCA. Likewise, the relatively low NPV of the KK test may be related to the low sensitivity of KK in this population.

One of the limitations of this study was the use of only three stool samples to evaluate the infection status by KK. Studies have demonstrated an increased sensitivity of KK with increasing numbers of stool samples evaluated; however, several studies only evaluated two KK, therefore, our study would have improved KK sensitivity over those^8^. Another limitation is that trace results were recorded as positive in the field study, based on the manufacturer’s instructions for CCA kit. The next study may consider trace results separately to better understand the range of CCA test performance. Nevertheless, to the best of our knowledge, this study is the first to assess the performance of CCA and KK, as well as the prevalence of *S. japonicum* infection, using LCA.

The decision regarding the treatment of trace positives should be related to the tolerance for false negatives versus false positives. In most clinical applications, if there is a large downside to a false negative (e.g., a trace called a negative), there are minimal risks to treatment, and one would usually opt to treat. In clinical scenarios, where missing or delaying a diagnosis is not dire and there is a risk of false positives, such as patient anxiety, additional testing burden, and cost, one might err on reduced sensitivity and better specificity. Given Praziquantel’s safety profile and tolerability, with side effects most profound among infected individuals, we would recommend higher sensitivity at the expense of some specificity in the context of schistosomiasis treatment. Employing various tests that complement each other has proven to be advantageous, particularly in low transmission communities, leading to more accurate diagnoses^42^. Further exploration into combining additional immunological diagnostic methods, such as the Circumoval Precipitin Test (COPT), could enhance the diagnostic platform for *S. japonicum* endemic regions, offering a more effective and practical approach^42,43^.

## Conclusions

In conclusion, the estimated sensitivity of CCA was higher than that of KK regardless of the age group, whereas the estimated specificity was higher using KK. Depending on the goals of testing, different approaches could be applied (a) at the community level to determine prevalence as a guide to MDA frequency or (b) individually to determine the need for treatment. However, the ease of use conferred by POC tests, such as POC-CCA, as well as their high sensitivity combined with a “low risk” of false positivity (lower specificity), make CCA an ideal approach. Its use together with MDA may increase the uptake among individuals who want to know their infection status before accepting treatment, although the cost remains a significant barrier to this approach^20,21^.

### Supplementary Information

Supplementary Information.

## Data Availability

The datasets generated and/or analyzed in the current study are available from a qualified researcher, Mario Jiz (mario.a.jiz@gmail.com), upon reasonable request.

## Abbreviations

CAA: Circulating anodic antigen
CCA: Circulating cathodic antigen
covSe: Covariance in sensitivity
covSp: Covariance in specificity
EITB: Electroimmunotransfer blot
ELISA: Enzyme-linked immunosorbent assay
epg: Eggs per gram
IB: Immunoblot
KK: Kato-Katz
LAMP: Loop-mediated isothermal amplification
LCA: Latent class analysis
MCMC: Markov Chain Monte Carlo
MDA: Mass drug administration
NPV: Negative predictive value
P: Probability
PCR: Polymerase chain reaction
POC-CCA: Point-of-care CCA test
PPV: Positive predictive value
prev: Prevalence
Se: Sensitivity
Sp: Specificity
STH: Soil-transmitted helminths
WB: Western blot
