# Supplementary material for: Evaluation of sensitivity and specificity of Kato-Katz and circulating cathodic antigen in terms of Schistosoma japonicum using latent class analysis
Source: Sci Rep. 2024 Apr 8;14:8164. doi: 10.1038/s41598-024-57863-9 (PMC11001968; doi:10.1038/s41598-024-57863-9)
Supplement: Supplementary file 1 — Supplementary Information. [file 41598_2024_57863_MOESM1_ESM.pdf]

Supplementary Methods. Code for running the LCA

```
install.packages("R2OpenBUGS")
install.packages("coda")
rm(list = ls())
   library(R2OpenBUGS)
          library(coda)
          library(lattice)
          library(dplyr)
   library(foreign)
select <- dplyr::select

#########LCA analysis (children)###########

setwd("C:/data/schistosoma1")
          data <- read.csv("01_Children.csv", na.strings ="", stringsAsFactors=F)
          tested <- as.vector(data$totaltested) # variable 'totaltested': number of subjects in each group
          result <- as.matrix(select(data, -site, -totaltested)) # Cleaned data (result) after deleting the following variables:
'site,' 'totaltested'
          LCA.data <- list(result = result, tested = tested)

##########    FUNCTION##################

# function 1: LCA.model
LCA.model <- function(){
   #====likelihood========================
        for (i in 1:12){
   ##KK+ and CCA +
        y[i, 4] <- prev[i] * (Se.KK * Se.CCA + c.Se.KK.CCA) + (1 - prev[i]) * ((1 - Sp.KK) * (1 - Sp.CCA) + c.Sp.KK.CCA)
   ##KK+ and CCA -
        y[i, 3] <- prev[i] * (Se.KK * (1 - Se.CCA) - c.Se.KK.CCA) + (1 - prev[i]) * ((1 - Sp.KK) * Sp.CCA - c.Sp.KK.CCA)
   ##KK- and CCA +
        y[i, 2] <- prev[i] * ((1 - Se.KK) * Se.CCA - c.Se.KK.CCA) + (1 - prev[i]) * (Sp.KK * (1 - Sp.CCA) - c.Sp.KK.CCA)
   ##KK- and CCA -
        y[i, 1] <- prev[i] * ((1 - Se.KK) * (1 - Se.CCA) + c.Se.KK.CCA) + (1 - prev[i]) * (Sp.KK * Sp.CCA + c.Sp.KK.CCA)
        result[i, 1 : 4] ~ dmulti(y[i, 1 : 4], tested[i])
        }

   #===prior distributions ========================================
   for (i in 1 : 12) {prev[i] ~ dbeta(1, 1) }
   Se.KK ~ dbeta(3.05, 1.15)
   Sp.KK ~ dbeta(21.20, 2.06)
   Se.CCA ~ dbeta(3.05, 1.15)
   Sp.CCA ~ dbeta(5.38, 1.49)

   c.Se.KK.CCA ~ dunif(L.c.Se.KK.CCA, U.c.Se.KK.CCA)
   c.Sp.KK.CCA ~ dunif(L.c.Sp.KK.CCA, U.c.Sp.KK.CCA)

   U.c.Se.KK.CCA <- min(Se.KK, Se.CCA)    - Se.KK * Se.CCA
   U.c.Sp.KK.CCA <- min(Sp.KK, Sp.CCA)    - Sp.KK * Sp.CCA

   L.c.Se.KK.CCA <- (Se.KK - 1)*(1 - Se.CCA)
   L.c.Sp.KK.CCA <- (Sp.KK - 1)*(1 - Sp.CCA)
```

```
}

# function 2: LCA.inits

LCA.inits <- function(){list(prev = runif(12, 0, 1),
                              Se.KK = runif(1, 0.2, 0.8),
                              Sp.KK = runif(1, 0.9, 1),
                              Se.CCA = runif(1, 0.4, 1),
                              Sp.CCA = runif(1, 0.6, 1),
                              c.Se.KK.CCA = 0,
                              c.Sp.KK.CCA = 0
)}

LCA.monitor <- c("prev",
                 "Se.KK", "Sp.KK",
                 "Se.CCA", "Sp.CCA",
                 "c.Se.KK.CCA",
                 "c.Sp.KK.CCA"
)


LCA.out11 <- bugs(LCA.data, LCA.inits, LCA.monitor, LCA.model,
                  n.iter=12000, n.burnin=2000, n.thin=25, n.chains=3, debug = T)

        # a. diagnosis
all(LCA.out11$summary[,"Rhat"] < 1.1)
min(LCA.out11$summary[,"n.eff"])
max(LCA.out11$summary[,"n.eff"])
        # b. Print results
print(LCA.out11, digits = 5)

# 2. Export """CSV""" dataset if all iterations
        outdset <- data.frame(LCA.out11$sims.list)
        write.csv (outdset, file= "C:/data/schistosoma1/01_Children_LCAresult3.csv")


#LCA analysis (adults)
#########################################################################
        data <- read.csv("02_Adults.csv", na.strings ="", stringsAsFactors=F)

        tested <- as.vector(data$totaltested) # variable 'totaltested': number of subjects in each group
        result <- as.matrix(select(data, -site, -totaltested)) # Cleaned data (result) after deleting the following variables: 
'site,' 'totaltested'
        LCA.data <- list(result = result, tested = tested)

# function 1: LCA.model
        LCA.model <- function(){
          #====likelihood========================
          for (i in 1:12){
            ##KK+ and CCA +
            y[i, 4] <- prev[i] * (Se.KK * Se.CCA + c.Se.KK.CCA) + (1 - prev[i]) * ((1 - Sp.KK) * (1 - Sp.CCA) + c.Sp.KK.CCA)
            ##KK+ and CCA -
            y[i, 3] <- prev[i] * (Se.KK * (1 - Se.CCA) - c.Se.KK.CCA) + (1 - prev[i]) * ((1 - Sp.KK) * Sp.CCA - c.Sp.KK.CCA)
```

```
        ##KK- and CCA +
        y[i, 2] <- prev[i] * ((1 - Se.KK) * Se.CCA - c.Se.KK.CCA) + (1 - prev[i]) * (Sp.KK * (1 - Sp.CCA) - c.Sp.KK.CCA)
        ##KK- and CCA -
        y[i, 1] <- prev[i] * ((1 - Se.KK) * (1 - Se.CCA) + c.Se.KK.CCA) + (1 - prev[i]) * (Sp.KK * Sp.CCA + c.Sp.KK.CCA)
        result[i, 1 : 4] ~ dmulti(y[i, 1 : 4], tested[i])          # observed data
      }

#===prior distributions ================================================
      for (i in 1 : 12) {prev[i] ~ dbeta(1, 1) }
      Se.KK ~ dbeta(3.05, 1.15)
      Sp.KK ~ dbeta(21.20, 2.06)
      Se.CCA ~ dbeta(3.05, 1.15)
      Sp.CCA ~ dbeta(5.38, 1.49)

      c.Se.KK.CCA ~ dunif(L.c.Se.KK.CCA, U.c.Se.KK.CCA)
      c.Sp.KK.CCA ~ dunif(L.c.Sp.KK.CCA, U.c.Sp.KK.CCA)


      U.c.Se.KK.CCA <- min(Se.KK, Se.CCA)    - Se.KK * Se.CCA
      U.c.Sp.KK.CCA <- min(Sp.KK, Sp.CCA)    - Sp.KK * Sp.CCA

      L.c.Se.KK.CCA <- (Se.KK - 1)*(1 - Se.CCA)
      L.c.Sp.KK.CCA <- (Sp.KK - 1)*(1 - Sp.CCA)
    }


    # function 2: LCA.inits

    LCA.inits <- function(){list(prev = runif(12, 0, 1),
                                 Se.KK = runif(1, 0.2, 0.8),
                                 Sp.KK = runif(1, 0.9, 1),
                                 Se.CCA = runif(1, 0.4, 1),
                                 Sp.CCA = runif(1, 0.6, 1),
                                 c.Se.KK.CCA = 0,
                                 c.Sp.KK.CCA = 0
    )}

    # function 3: LCA.monitor, just monitoring variables and not calculations for diagnostics
    LCA.monitor <- c("prev",
                     "Se.KK", "Sp.KK",
                     "Se.CCA", "Sp.CCA",
                     "c.Se.KK.CCA",
                     "c.Sp.KK.CCA"
    )


    LCA.out11 <- bugs(LCA.data, LCA.inits, LCA.monitor, LCA.model,
                      n.iter=12000, n.burnin=2000, n.thin=25, n.chains=3, debug = T)

    # a. diagnosis
    all(LCA.out11$summary[,"Rhat"] < 1.1)
    min(LCA.out11$summary[,"n.eff"])
    max(LCA.out11$summary[,"n.eff"])
```

```
	# b. Print results
	print(LCA.out11, digits = 5)

	# 2. Export """CSV""" dataset if all iterations
	outdset <- data.frame(LCA.out11$sims.list)
	write.csv (outdset, file= "C:/data/schistosoma1/02_Adults_LCAresult3.csv")



#end of first analysis
#compute weighted infection prevalence and average of KK and CCA sensitivity and specificity. It will be used in the second analysis.


#second analysis

	prev_PPV_NPV <- function(iter, Ch_n, Ch_KK_prev, Ch_CCA_prev, Ch_prev, Ch_KK_se, Ch_CCA_se, Ch_KK_sp, Ch_CCA_sp,		Ad_n, Ad_KK_prev, Ad_CCA_prev, Ad_prev, Ad_KK_se, Ad_CCA_se, Ad_KK_sp, Ad_CCA_sp)
		{dataset=data.frame(matrix(0, nrow=30000, ncol=14))
		dataset[,1]=rbinom(n = iter, size = Ch_n, Ch_prev) ; overall_prev=Ch_prev
		dataset[,2]=rbinom(n = iter, size = Ch_n, Ch_KK_prev) /Ch_n; KK_se=Ch_KK_se; KK_sp=Ch_KK_sp; KK_PPV=(overall_prev*KK_se)/((overall_prev*KK_se)+(1-overall_prev)*(1-KK_sp));		KK_NPV=(1-overall_prev)*KK_sp/((overall_prev*(1-KK_se))+(1-overall_prev)*KK_sp)
		dataset[,3]=rbinom(n = iter, size = Ch_n, KK_PPV) /Ch_n
		dataset[,4]=rbinom(n = iter, size = Ch_n, KK_NPV) /Ch_n
		dataset[,5]=rbinom(n = iter, size = Ch_n, Ch_CCA_prev) /Ch_n; CCA_se=Ch_CCA_se; CCA_sp=Ch_CCA_sp;		CCA_PPV=(overall_prev*CCA_se)/((overall_prev*CCA_se)+(1-overall_prev)*(1-CCA_sp)); CCA_NPV=(1-overall_prev)*CCA_sp/((overall_prev*(1-CCA_se))+(1-overall_prev)*CCA_sp)
		dataset[,6]=rbinom(n = iter, size = Ch_n, CCA_PPV) /Ch_n
		dataset[,7]=rbinom(n = iter, size = Ch_n, CCA_NPV) /Ch_n
		dataset[,8]=rbinom(n = iter, size = Ad_n, Ad_prev) ; overall_prev=Ad_prev
		dataset[,9]=rbinom(n = iter, size = Ad_n, Ad_KK_prev) /Ad_n; KK_se=Ad_KK_se; KK_sp=Ad_KK_sp; KK_PPV=(overall_prev*KK_se)/((overall_prev*KK_se)+(1-overall_prev)*(1-KK_sp));		KK_NPV=(1-overall_prev)*KK_sp/((overall_prev*(1-KK_se))+(1-overall_prev)*KK_sp)
		dataset[,10]=rbinom(n = iter, size = Ad_n, KK_PPV) /Ad_n
		dataset[,11]=rbinom(n = iter, size = Ad_n, KK_NPV) /Ad_n
		dataset[,12]=rbinom(n = iter, size = Ad_n, Ad_CCA_prev) /Ad_n; CCA_se=Ad_CCA_se; CCA_sp=Ad_CCA_sp;		CCA_PPV=(overall_prev*CCA_se)/((overall_prev*CCA_se)+(1-overall_prev)*(1-CCA_sp)); CCA_NPV=(1-overall_prev)*CCA_sp/((overall_prev*(1-CCA_se))+(1-overall_prev)*CCA_sp)
		dataset[,13]=rbinom(n = iter, size = Ad_n, CCA_PPV) /Ad_n
		dataset[,14]=rbinom(n = iter, size = Ad_n, CCA_NPV) /Ad_n
	out<<-dataset}

prev_PPV_NPV (iter=30000, Ch_n=484, Ch_KK_prev=0.502066116, Ch_CCA_prev=0.898760331, Ch_prev=0.633545756, Ch_KK_se=0.660, Ch_CCA_se=0.948, Ch_KK_sp=0.781, Ch_CCA_sp=0.215,
					Ad_n=572,	Ad_KK_prev=0.318181818,	Ad_CCA_prev=0.667832168, Ad_prev=0.595696921, Ad_KK_se=0.436, Ad_CCA_se=0.864, Ad_KK_sp=0.855, Ad_CCA_sp=0.628)

Result2=out;
colnames(Result2)=c("Children_prev","Children_KK_prev","Children_KK_PPV","Children_KK_NPV","Children_CCA_prev","Children_CCA_PPV","Children_CCA_NPV","Adults_prev","Adults_KK_prev","Adults_KK_PPV","Adults_KK_NPV","Adults_CCA_prev","Adults_CCA_PPV","Adults_CCA_NPV")
```

```
prev_ppv_npv=cbind(Result2)
write.csv (prev_ppv_npv, file= "C:/data/schistosoma1/second_analysis_result3.csv")
```
